# Polysaccharides: Nature’s Guardians of Freshness in Food Preservation

**DOI:** 10.3390/molecules31091545

**Published:** 2026-05-06

**Authors:** Amanullah Sabir, Sadaqat Ali, Muhammad Zubair Khalid, Ashoka Shankarappa, V. J. Sangeetha, Samreen Ahsan, Anand Kumar, Kamran, Kit-Leong Cheong, Saiyi Zhong

**Affiliations:** 1 Guangdong Provincial Key Laboratory of Aquatic Product Processing and Safety, College of Food Science and Technology, Guangdong Ocean University, Zhanjiang 524088, China; amaanullahsabir@gmail.com (A.S.); drsadaqat6@gmail.com (S.A.); anandkumarfoodtech11@gmail.com (A.K.); kamranniamatali@outlook.com (K.); 2 Department of Food Science, Faculty of Life Sciences, Government College University, Faisalabad 38000, Pakistan; zubairkhalid730@gmail.com; 3 Department of Food Science and Nutrition, College of Agriculture, University of Agricultural Sciences, Mandya 571405, India; ashokafsn@gmail.com; 4 Department of Nutrition, Osmania University, Hyderabad 500007, India; sangeethavj04@gmail.com; 5 Institute of Food Science and Technology, Faculty of Food, Health Science and Technology, Khwaja Fareed University Engineering and Information Technology, Rahim Yar Khan 64200, Pakistan; samreenahsan@kfueit.edu.pk

**Keywords:** polysaccharides, food preservation, edible films and coatings, antioxidant activity, antimicrobial activity, natural packaging, shelf-life extension

## Abstract

Polysaccharides are structurally diverse biopolymers composed of multiple monosaccharide units linked through glycosidic bonds. Their complexity, biodegradability, and functional versatility make them integral to biological systems as well as modern industrial application. Sourced from plants, fungi, marine organisms, animals, and microbes, these natural polymers exhibit a broad spectrum of bioactivities, including antioxidant, antimicrobial, immunomodulatory, and physicochemical protective functions. In the context of food preservation, polysaccharides have gained significant attention as sustainable alternatives to synthetic preservatives and conventional packaging materials. This review summarizes the classification and structural attributes of polysaccharides that influence their functional performance, particularly their ability to scavenge free radicals, inhibit foodborne pathogens, and form protective barrier systems. Special emphasis is placed on their use in edible films, coatings, and encapsulation systems that enhance the shelf life of fruits, vegetables, meats, dairy, beverages, and bakery products. Challenges related to stability, sensory impact, and regulatory compliance are also discussed. Overall, polysaccharides demonstrate substantial potential as eco-friendly, bioactive packaging agents and controlled-release carriers, contributing to safer, greener, and more sustainable food preservation technologies.

## 1. Introduction

Polysaccharides are high molecular weight biopolymers composed of repeating monosaccharide units linked by glycosidic bonds. They represent one of the four major classes of biomacromolecules, alongside proteins, nucleic acids, and lipids, and are ubiquitous in animals, plants, algae, fungi, and microorganism’s polymers exhibit remarkable structural diversity, including variations in monosaccharide composition, linkage types, branching patterns, molecular weight, and functional groups. Such structural versatility underpins their broad functional capabilities, ranging from structural support, energy storage, and cell signalling in living organisms to industrial and biotechnological applications in food, pharmaceuticals, and environmental technologies [1].

In food science, polysaccharides have gained significant attention due to their biodegradability, biocompatibility, renewability, and multifunctional properties [2,3,4].They can form edible films and coatings that act as physical barriers to moisture and gas transfer, serve as carriers of bioactive agents, and function as matrices with antimicrobial and antioxidant properties, thereby enhancing the shelf life and quality of perishable foods [1,5]. Beyond preservation, polysaccharides offer potential in active and intelligent packaging systems by incorporating bioactive compounds, such as polyphenols, essential oils, and nanofillers, to enhance barrier performance and functional efficacy [6,7].

The increasing reliance on synthetic preservatives and petroleum-based packaging has raised significant environmental and health concerns. Conventional packaging materials often exhibit limited biodegradability, persistence in ecosystems, and potential toxicity, which has led to stricter regulations and heightened consumer demand for safer, eco-friendly alternatives [8,9]. Consequently, polysaccharide-based films and coatings have emerged as promising solutions that align with sustainable food technology goals, offering natural, safe, and multifunctional strategies for food preservation [10].

Polysaccharides also exhibit a range of bioactive functions that extend beyond structural and barrier roles. Natural polysaccharides derived from plants, algae, and microorganisms have demonstrated antioxidant, antimicrobial, immunomodulatory, and metabolic regulatory activities, which are often link to their molecular weight, chain conformation, and specific chemical structures [11,12]. For instance, arabinogalactans from *Chlorella pyrenoidosa* have shown that higher molecular weight variants exhibit stronger immunostimulatory activity [13]. The structure–function relationship of polysaccharides remains a key focus of research, as hyperbranched structures, high molecular weights, and aggregation tendencies can influence their biological and functional properties [14].

In the context of sustainable packaging, polysaccharides derive from plant and marine biomass or produce via microbial fermentation, and represent abundant and renewable resources. Their water-soluble forms are particularly attractive for generating natural antioxidants and bioactive agents, which can improve food quality while reducing environmental impact [15,16]. Compared to proteins and nucleic acids, polysaccharides are more stable under heat and processing conditions, further enhancing their suitability for materials science and food applications [17]. Polysaccharides act as wide range of functional biopolymers in developing effective active packaging system to modify food preservation. These properties of polysaccharides are associated with their renewability, structural diversity, and biocompatibility. They also act as good carrier and responsible for controlled release of bioactive moieties by controlling stimuli mechanism responses such as enzyme and pH. However, the functionality of polysaccharides is mainly linked between their structure and their function. Structural parameters of polysaccharide depend mainly on the degree of polymerization and branching, molecular weight, monomer composition, crystallinity, charge density, and the presence of functional groups e.g., carboxylic acid group, hydroxyl, sulfate, and amino groups play a key role in determining physicochemical and biological properties [18]. These functional properties of polysaccharide directly influence their mechanical strength and film-forming property, and they act as barrier against gas and moisture, antimicrobial and antioxidant activity, and interactions with food matrices or active compounds [19].

**Figure 1:**
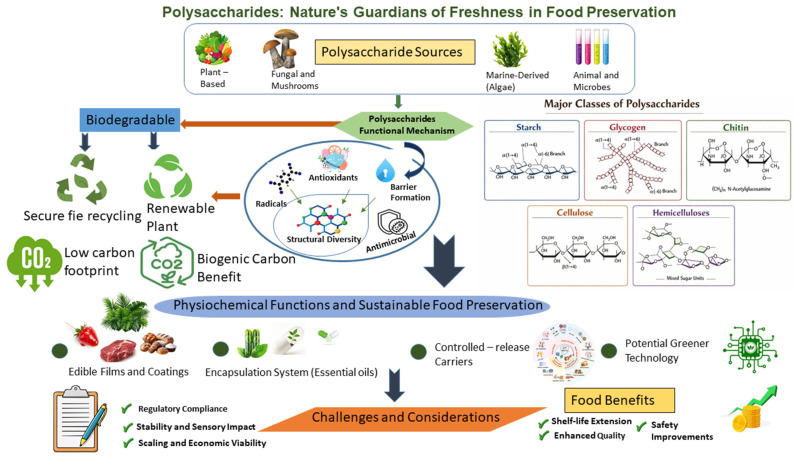
Summarization of polysaccharides: nature’s guardians of freshness in food preservation.

The scope of this review encompasses a comprehensive examination of polysaccharides in food preservation, highlighting their sources, classification, structural diversity, and functional attributes. It is mainly emphasizing a structure–function-oriented framework to analyze polysaccharides from various sources including plant, microbial, marine, and animal, and it is making a link between their molecular architecture and their performance in encapsulation systems, edible films, coatings, and active food packaging applications. Emphasis is placed on their antioxidant, antimicrobial, and barrier properties, along with their application in edible films, coatings, and active packaging systems. Challenges such as compatibility, processing limitations, and regulatory issues are discussed, and future perspectives are provided. This synthesis underscores the pivotal role of polysaccharides as nature’s guardians of freshness in modern food preservation strategies. Overall, this manuscript revolves around Figure 1.

## 2. Sources, Types, and Composition of Functional Polysaccharides

Functional polysaccharides are derived from a diverse range of natural biological sources, each offering distinctive structural features and physicochemical properties that are highly relevant to food preservation and active packaging. Their natural abundance across plants, fungi, marine biomass, animals, and microbial fermentation systems positions them as versatile and sustainable alternatives to conventional synthetic materials. The increasing demand for bio-based, biodegradable, and functional ingredients in the food sector has further accelerated interest in these polysaccharides as key contributors to clean-label and eco-friendly preservation strategies [20,21].

### 2.1. Plant-Based Polysaccharides

Plant-derived polysaccharides constitute one of the largest and most exploited categories of natural biopolymers due to their biodegradability, sustainability, low production cost, and wide natural availability. They encompass structurally diverse macromolecules such as pectin, cellulose, starch, plant gums, and arabinogalactans each possessing unique chemical architectures that determine their functional roles in food systems [22]. The exploration of novel plant sources and extraction technologies continues to expand their applicability, particularly in food preservation and packaging.

Pectin, primarily sourced from citrus peels and apple pomace, is widely utilized for its excellent gelling, stabilizing, and film-forming properties. Its ability to form strong oxygen-barrier films and serve as a carrier for natural antimicrobials and antioxidants makes it an effective component of edible coatings and active packaging materials [21,23].

Cellulose and its nanostructured derivatives, especially nanocellulose, have gained significant attention for their exceptional mechanical strength, biodegradability, and improved water and gas barrier properties. These attributes make cellulose-based materials promising candidates for next-generation biodegradable packaging films [20,24].

Starch is one of the most abundant renewable polysaccharides and remains a major focus in the development of bio-based packaging materials. Although native starch exhibits limitations such as brittleness and moisture sensitivity, recent research highlights the effectiveness of blending starch with plasticizers, biopolymers, or nanofillers to enhance flexibility, barrier properties, and overall stability [20,23].

Plant gums, including guar gum (*Cyamopsis tetragonolobus*) seeds and locust bean gum (*Ceratonia silique*), along with arabinogalactans obtained from seeds and herbal tissues, contribute valuable thickening, emulsifying, and film-forming functionalities. Many of these gums also exhibit antioxidant and prebiotic activities, offering additional benefits when incorporated into functional coatings [24]. Guar gum is excellent for polysaccharide use in packaging material due to its biodegradable and hydrophobic properties. It is whitish in color and does not impart any color onto packaging material. It also has good antimicrobial traits and due to its mechanical strength, it keeps the product mechanically safe [25].

Moreover, use of locust bean gum in different edible coating formulations has reported to extend shelf life of fruits, vegetables, and it also shows potential application in meat-based products. The incorporation of locust bean gum reduces syneresis and stabilizes freeze thawing in starch-based formulations [26]. Likewise, edible coating with flaxseed gum in fat-based products is helpful to reduce the oxidation of lipid and development of rancidity [27]. Carrageenan is also very effective to fabricate edible coatings for perishable food commodities. It modifies the stability of emulsions, viscosity, and improves the coating property [28].

Recent advancements emphasize the development of multifunctional plant-derived polysaccharide films enriched with essential oils, antioxidants, nanoparticles, and bioactive compounds to create packaging systems with enhanced antimicrobial, antioxidative, and shelf-life-extending capabilities. These innovations reinforce the role of plant polysaccharides as foundational materials for sustainable and intelligent food preservation solutions [21,23]. Moreover, the variation in raw materials due to differences in species, cultivar, growth conditions, harvest time, and geographical origin can lead to substantial differences in molecular weight, monosaccharide composition, and degree of substitution. In addition, extraction and purification methods (e.g., solvent type, temperature, pH, enzymatic treatment) strongly influence polysaccharide yield, structural integrity, and functional group availability. These factors contribute to pronounced structural heterogeneity, which in turn affects reproducibility, functional performance, and cross-study comparability. Such variability remains a major challenge for standardization, scalability, and industrial implementation of polysaccharide-based food preservation systems [29].

### 2.2. Fungal and Mushroom Polysaccharides

Fungi and edible mushrooms represent an important and growing source of functional polysaccharides with diverse structural features and significant biological activity. These polysaccharides originating from both fungal cell walls and extracellular secretions have gained increasing attention due to their multifunctionality and broad applicability in food, biomedical, cosmetic, and pharmaceutical fields [30]. Major fungal biopolymers include chitin, β-glucans, mannoproteins, and a range of extracellular polysaccharides (EPS) such as pullulan, scleroglucan, and schizophyllan, each offering distinct techno-functional and bioactive properties.

Chitin-based polymers and their derivatives, such as chitosan, chitin–glucan complexes (CGC), and chitosan–glucan complexes (ChGC), have been widely investigated for their antimicrobial, film-forming, and biocompatible properties, supporting their use in food preservation and active packaging applications [31]. Over decades of research, fungal-derived mannans, glucans, and chitinous polymers have been isolated from a variety of fungal genera, forming a rich library of bioactive polysaccharides with applications highlighted across food, medical, and industrial sectors.

Among these, β-glucans particularly from *Saccharomyces cerevisiae* and edible mushrooms are the most extensively studied due to their potent immunomodulatory, antioxidant, and antimicrobial functions [32]. Their incorporation into edible films enhances biological activity and improves the protective performance of packaging materials against oxidative degradation and microbial spoilage.

Similarly, galactomannans, produced by fungi and found in certain seeds, offer excellent rheological properties, contributing to viscosity, gel strength, and water-binding capacity. These attributes make them valuable natural stabilizers, thickeners, and texture modifiers in food formulations [33].

Recent advances in fungal biotechnology emphasize the valorization of mushroom biomass and fungal mycelia for sustainable packaging. Fungal polysaccharides explored as structural components in biodegradable films and coatings, leveraging their innate antimicrobial and antioxidative properties to enhance food quality and extend shelf life [34].

Overall, fungal and mushroom polysaccharides present a versatile and renewable platform for developing functional ingredients and eco-friendly preservation systems, further strengthening their relevance within emerging circular and bio-based food technologies.

### 2.3. Marine-Derived Polysaccharides

Marine ecosystems, particularly seaweeds, represent one of the richest reservoirs of structurally diverse and bioactive polysaccharides. Marine algae contain not only polysaccharides but also proteins, peptides, lipids, amino acids, polyphenols, and essential minerals, making them valuable functional ingredients for food and biotechnological applications [35]. Based on pigmentation and biochemical composition, seaweeds are classified into three major groups: green algae (Chlorophyceae), red algae (Rhodophyceae), and brown algae (Phaeophyceae). These seaweeds have incorporated into traditional Asian diets in soups, salads, snacks, and condiments, reflecting their nutritional significance and broad culinary use [15].

Marine-derived polysaccharides such as alginate, carrageenan, agar, and fucoidan are particularly notable for their film-forming capabilities and biological functionalities. Alginate, extracted primarily from brown seaweeds, is widely applied in edible coating technologies due to its strong gelation ability, biocompatibility, and capacity to encapsulate antimicrobial or antioxidant compounds. Despite these advantages, alginate-based films often require blending with other biopolymers to improve water resistance and mechanical stability [36].

Carrageenan and agar, obtained from red algae, are highly effective gelling agents and are commonly used in biodegradable films and intelligent packaging systems. Their excellent texture-forming properties, thermal stability, and compatibility with bioactive substances make them integral components of sustainable packaging innovations [37].

Fucoidan, a sulfated polysaccharide abundant in brown seaweeds, has gained increasing attention due to its robust antioxidant, antiviral, and antimicrobial activities. These properties position fucoidan as a promising functional ingredient for active food preservation systems and antioxidant-enriched coatings [38].

Overall, marine polysaccharides are emerging as key materials in the development of bioactive coatings, antioxidant films, edible hydrogels, and nanocomposite packaging. Their natural abundance, renewability, and multifunctionality support global efforts toward ocean-derived biopolymers and sustainable food preservation technologies [36,37].

### 2.4. Animal and Microbial Polysaccharides

Animal and microbial-derived polysaccharides constitute another important class of functional biopolymers with significant applications in food preservation and packaging. Among these, chitosan, hyaluronic acid, and microbial gums such as xanthan gum are the most prominent due to their bioactivities, structural versatility, and technological functionality.

Chitosan, obtained primarily from crustacean shell chitin or produced through fungal fermentation, is one of the most widely investigated natural polymers in food preservation. Its intrinsic antimicrobial activity, film forming capability, and compatibility with natural antioxidants and antimicrobials make it highly suitable for active packaging. Chitosan-based films enriched with essential oils, phenolic extracts, or nanoparticles have demonstrated strong potential for extending the shelf life of fruits, vegetables, seafood, and meat products by inhibiting microbial growth and oxidative deterioration [39,40].

Hyaluronic acid, predominantly produced via microbial fermentation, has recently attracted attention as a functional edible coating due to its excellent water-binding capacity, moisture retention, and emerging antioxidant properties. These attributes enable its application in fresh produce coatings and high-moisture foods to reduce dehydration and maintain textural quality [41].

Xanthan gum, an extracellular polysaccharide synthesized by *Xanthomonas campestris*, is widely used in the food industry for its stabilizing, thickening, and viscosity-modifying properties. Recent advancements highlight its incorporation into biodegradable films and composite coatings to enhance elasticity, mechanical strength, and handling characteristics, thereby supporting its role in eco-friendly packaging systems [42].

Microbial fermentation provides a controlled and sustainable platform for producing polysaccharides with desired molecular weights and tailored functionalities, ensuring batch-to-batch consistency and suitability for industrial-scale edible films and packaging applications [40].

Across plant, fungal, marine, animal, and microbial sources, polysaccharides contribute diverse structures and functionalities that underpin their value in modern food preservation technologies. Their biodegradability, rheological functionality, film-forming behavior, and inherent antimicrobial or antioxidant activities position them as indispensable materials for next-generation edible films, active coatings, and biocompatible packaging solutions [20,43].

Table 1 summarizes the various sources of functional polysaccharides, their structural types, monosaccharide composition, taxonomic classification, and key references from recent studies.

## 3. Classification of Polysaccharide

Polysaccharides are high-molecular-weight carbohydrates composed of repeating monosaccharide units linked through glycosidic bonds. They play essential biological roles across living systems, functioning as structural components (e.g., cellulose in plants, chitin in arthropods), energy reserves (e.g., starch in plants, glycogen in animals), and mediators of cell signaling, immune modulation, and intercellular recognition. Examples such as peptidoglycan in bacterial cell walls and chitin in fungal cell walls illustrate the structural diversity and functional complexity of these biomacromolecules. Their diverse architectures allow polysaccharides to participate in a wide range of biological mechanisms, maintaining vital physiological roles across species [87].

The following subsections describe major classes of biologically relevant polysaccharides, their structures, and functional characteristics [88,89,90,91].

Polysaccharides classification can be conducted in three different categories, including

(i) Biological source (plant, animal, microbial),
(ii) Structural Polysaccharide (Cellulose, Hemicellulose, and Chitin)

Overall, Table 2 provides a comparative overview of polysaccharides from different sources including plant, marine, and microbial or animal sources, highlighting their structure function relationships in bioactive packaging applications. Table 2 differentiates key performance markers such as mechanical strength of film-forming ability, its antioxidant or antimicrobial functionality, and barrier behavior. It is also focusing on quantitative and semi-quantitative measurements along with discussing on limitation and variability of active film made from different polysaccharide and how their structure effects on food performance.

### 3.1. Classification Based on Biological Function

#### 3.1.1. Storage Polysaccharides

##### Starch

Starch is the major storage polysaccharide in plants and is abundant in cereals, legumes, tubers, and seeds. Structurally, starch consists of glucose monomers connected through α-1, 4-glycosidic bonds, forming two main fractions: amylose, a mostly linear polymer, and amylopectin, a highly branched polymer with α-1, 6 linkages at branch points [100]. As reported by [100], starch is the most predominant carbohydrate in grains, contributing approximately 4 kcal/g upon digestion in the human gastrointestinal tract. Although both amylose and amylopectin are composed of D-glucose, their molecular weight, branching pattern, and chain length distribution results in significant differences in physicochemical behaviour, including gelatinization, retrogradation, and gel strength [101,102].

Native starch often exhibits limitations such as weak thermal stability and susceptibility to shear, making it unsuitable for many industrial applications in its unmodified form [103]. To enhance functionality, starch is frequently modified through physical, enzymatic, or chemical methods. One example is octenyl succinic anhydride (OSA) starch, produced by esterifying starch with OSA to introduce amphiphilic properties that improve emulsification performance [104].

Starch remains a key energy source in plant tissues such as cereal grains, potatoes, and other starchy crops. However, starch functionality could also be augmented through natural modification which is also known as bio-based modification [105]. Natural modification is conducted by blending starch with natural modifiers for example with dietary fibers, gums, essential oils, protein or by incorporating plant extracts, plant mucilage, and fiber-based fillers (cellulose), which have been shown to modify barrier properties, tensile strength, and antimicrobial activity of starch-based films [106].

##### Glycogen

Glycogen is the primary glucose storage polysaccharide in animals and exhibits a highly branched structure, with α-1,4-glycosidic bonds forming linear chains and α-1,6 linkages creating frequent branching points. This dense branching allows for rapid mobilization of glucose when the body requires energy, particularly in liver and muscle tissues [107].

Animal-derived structural polysaccharides also include glycosaminoglycans, which consist of alternating uronic acids and N-acetylhexosamines linked through β-1,4 and β-1,3 bonds. Examples include hyaluronic acid, keratan sulfate, and heparin. These polymers contain variable sulfate groups that influence charge density and functionality. Although glycogen is primarily associated with carbohydrate storage, these related animal polysaccharides demonstrate the broader structural diversity observed in biological systems.

#### 3.1.2. Structural Polysaccharides

##### Cellulose

Cellulose is the most abundant structural polysaccharide in nature and a major component of plant cell walls. It consists of linear chains of β-D-glucose linked through β-1,4-glycosidic bonds, forming tightly packed, crystalline microfibrils that provide rigidity and mechanical strength to plants. In humans, cellulose functions as an indigestible dietary fiber [108].

Cellulose can be extracted from cereal bran such as wheat, rice, and oats using acidic or alkaline treatment. Chemical modification yields derivatives such as carboxymethyl cellulose (CMC), produced by etherification with monochloroacetic acid, resulting in improved water solubility. Another widely used form is microcrystalline cellulose (MCC), obtained from α-cellulose through controlled acid hydrolysis [109].

Although cellulose is not commonly used in meat products, functional studies show that MCC can enhance the structural integrity and texture of products like beef patties, while excessive CMC may impair protein network formation and reduce sensory quality [110]. Beyond food applications, cellulose is also the primary component of cotton fibers.

##### Hemicellulose

Hemicellulose comprises a heterogeneous group of polysaccharides present in plant cell walls, accounting for approximately 20–30% of plant biomass [111]. Unlike cellulose, hemicelluloses are branched and contain various monosaccharides, including xylose, arabinose, mannose, galactose, glucose, and glucuronic acid [87]. The backbone predominantly consists of β-(1→4) linkages, whereas branching patterns vary based on source and type.

Major hemicellulose types include xylans, arabinoxylans, glucomannans, β-glucans, and xyloglucans, each contributing differently to structural and functional characteristics in plant tissues [112]. Extraction typically involves neutral or alkaline treatments, sometimes supported by solvents such as dimethyl sulfoxide or combinations such as hydrogen peroxide–alkali or alkali–chlorite systems [113].

**Figure 2:**
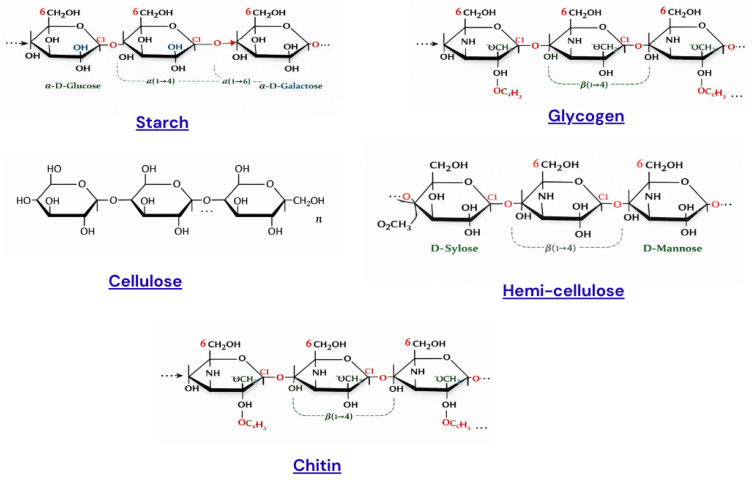
Representative structural models of major polysaccharides used in food and biomaterial applications, including chitin, cellulose, starch.

Hemicelluloses are recognized for their water-holding ability, prebiotic activity, emulsification properties, and potential use in biodegradable packaging materials and edible films, making them valuable in food technology and sustainable biomaterial development. Table 3 presents polysaccharides along with their biological source, key functional properties for food preservation, and applications in bioactive packaging. The structural variations among major polysaccharides are depicted in Figure 2.

##### Chitin

Chitin is a structural biopolymer composed of N-acetyl-D-glucosamine units linked through β-1,4-glycosidic bonds. It is abundant in the exoskeletons of arthropods (e.g., crustaceans, insects) and in the cell walls of fungi. Chitin provides rigidity, protection, and flexibility, enabling organisms to resist pathogens and environmental stressors.

Chitin derived chitosan, obtain through deacetylation and has gain significant interest due to its solubility under acidic conditions, biological compatibility, and functional groups (hydroxyl, amino, and acetamido) that enable diverse chemical modifications [114]. Chitin/chitosan-based formulations are use in oral drug delivery to reduce cholesterol and blood pressure, as well as in ophthalmic preparations to improve retention and bioavailability of therapeutics [115].

Chemical modification methods such as etherification, esterification, grafting, quaternization, and Schiff-base formation enhance chitosan’s (derivative of chitin) reactivity, water solubility, and functional properties, enabling applications in drug delivery, antimicrobial films, and targeted delivery systems [116]. Chitin thus represents an essential structural polysaccharide with broad biomedical and food-related applications [117,118].

## 4. Functional Bioactivities and Mechanisms of Polysaccharide-Mediated Food Preservation

Polysaccharides obtained from natural sources such as plants, fungi, algae, animals, and microbes have attracted increasing attention as multifunctional agents in food preservation. Their effectiveness arises from the integration of biological bioactivities primarily antioxidant and antimicrobial effects, with physicochemical functionalities such as film formation, encapsulation, and textural stabilization. Together, these properties enable polysaccharides to control oxidative degradation, inhibit microbial proliferation, and maintain food quality and safety during storage, aligning well with clean-label and sustainable preservation strategies [20,21].

### 4.1. Antioxidant Bioactivity and Oxidative Stability

Polysaccharide shows intrinsic antioxidant mechanisms due to their chemical structure having a hydroxyl group (-OH), so they can donate electron or hydrogen atom to remove free radicals. They also have carboxyl, sulfate, uronic acid, and amino group that have the ability to donate electrons and stabilize radicals. Their functional groups can bind metal ions like (Cu^2+^ and Fe^2+^) that act as pro-oxidant and prevent lipid oxidation [88].

The Nrf2/ARE pathway regulates the activity of antioxidant enzymes. Important enzymes that protect against dangerous free radicals, including SOD, CAT, and GSH-Px, are involved in this route [119]. By activating Nrf2, ginseng and other polysaccharides increase the expression of certain enzymes, hence enhancing their antioxidant ability [77]. According to studies, polysaccharides from a variety of plants, including *Astragalus* and *Sargassum fusiforme*, activate Nrf2, strengthening the antioxidant effects and preventing oxidative stress. Protecting against oxidative damage and preserving cellular homeostasis depend on this process [120].

Antioxidant enzymes like SOD, CAT, and GSH-Px help neutralize free radicals and transform them into non-toxic molecules [121]. Polysaccharides are involved in the efficient scavenging of free radicals and the regulation of antioxidant enzymes [122]. Research indicates that polysaccharides derived from diverse sources possess potent antioxidant characteristics that eliminate detrimental free radicals such as superoxide and hydroxyl anions [79].

Oxidative reactions are a major cause of food deterioration, resulting in lipid peroxidation, protein oxidation, discoloration, nutrient loss, and the development of off-flavours. Natural polysaccharides exhibit antioxidant activity through complementary mechanisms that collectively enhance oxidative stability in food systems.

Many polysaccharides can directly scavenge reactive oxygen species (ROSs) and other free radicals, including DPPH, ABTS, hydroxyl, and superoxide radicals. Their abundant hydroxyl, carboxyl, sulfate, and amino functional groups facilitate hydrogen or electron donation, thereby neutralizing reactive species and interrupting oxidative chain reactions. Structural attributes such as lower molecular weight, higher solubility, and increased exposure of reactive groups enhance radical scavenging efficiency, while chemical modifications such as sulfation, phosphorylation, and acetylation further improve antioxidant performance [123,124,125].

Beyond direct radical quenching, polysaccharides can modulate endogenous antioxidant defence systems through the nuclear factor erythroid 2–related factor 2 (Nrf2)/antioxidant response element (ARE) signalling pathway. Activation of Nrf2 promotes its dissociation from Keap1 and upregulates downstream antioxidant enzymes, including superoxide dismutase, catalase, and glutathione peroxidase. Although this mechanism is primarily characterized in biological systems, it is increasingly relevant to food preservation through stabilization of bioactive components, suppression of oxidative enzymes, and protection of sensitive nutrients in functional and minimally processed foods [14].

Certain polysaccharides also regulate the activity of endogenous antioxidant and pro-oxidant enzymes, enhancing oxidative balance by suppressing enzymes such as inducible nitric oxide synthase (iNOS). These indirect effects further reduce ROS accumulation and contribute to improved oxidative stability during storage.

The antioxidant activity documented to polysaccharides need careful explanation, as it may come from intrinsic structural features of the polysaccharide backbone or from phenolic that are attributed to co-extracted low-molecular-weight compounds including, flavonoids and pigments and sometimes secondary metabolites. In extraction of polysaccharide from an algal, fungal, or plant source, incomplete purification may result in residual impurities that considerably contribute to radical scavenging activity [126]. Likewise, intrinsic antioxidant activity of polysaccharides is their structural characteristics, including degree of branching, molecular weight, composition of monosaccharide, and the presence of functional groups such as carboxyl, sulfate, hydroxyl and amino groups. These structural parameters contribute to antioxidant values by the donation of a hydrogen atom from functional group hydrogen atom donation, transfer of electrons, and chelation of metal ions that inhibit oxidative reactions. In contrast, residues from phenolic compound arises antioxidant activity that is stronger and rapid and that is because of conjugated aromatic structure of phenolic compounds [1].

Collectively, these mechanisms demonstrate that polysaccharides act as effective natural antioxidants by combining free radical scavenging, enzyme regulation, and signalling modulation, thereby delaying oxidative degradation and extending shelf life. Moreover, antioxidant effects may be overestimated because of rigorous purification and compositional analysis, and imperfectly recognised to the polysaccharide backbone.

### 4.2. Antimicrobial Bioactivity and Microbial Control

Polysaccharides can act as antimicrobial through their biological and physicochemical interaction. Their surface charge plays very important role in this mechanism for example, positively charged amino group of chitosan interact with the negative charge microbial cell membrane and causing disruption of microbial cell wall. This leads to leakage of intracellular contents and ultimately results in cell death [89]. Crystallinity in their structure provides control of gasses (O_2_, CO_2_) and barrier aroma properties by limiting molecular mobility. Likewise, different functional groups (–OH, –COOH, –NH_2_, –SO_4_) impart antioxidant activity and play a role as a metal chelating agent and interact with other bioactive compounds. The difference in charge creates an electrostatic charge with the microbial cell membrane so that the microbial cell disrupts and the product shelf life is extended [90].

Polysaccharide coatings act as strong barrier by forming a protective layer on food surfaces, preventing microbial attachment and their colonization [88]. Polysaccharides act as strong barrier for gasses like (O_2_ and CO_2_) by restricting diffusion of gasses due to dense hydrogen-bonded networks. Moreover, crystalline structures like those in cellulose decrease free volume in films [91]. Overall, the molecular weight of polysaccharides determines the integrity of films, mechanical strength, and gas diffusion resistance, as well as the degree of branching influence on solubility of edible film matrix and film flexibility [90].

Microbial spoilage and foodborne pathogens remain major challenges in food safety and shelf-life extension. Many natural polysaccharides exhibit intrinsic antibacterial and antifungal activity or serve as carriers that enhance antimicrobial efficacy in food systems.

Recent research highlights the diverse biological benefits of plant polysaccharides, serving as antibiotics and antioxidants [127]. These polysaccharides, sourced from edible materials such as olive leaves, *Diaphragma juglandis*, *Broussonetia papyrifera* fruits, and *Malva aegyptiaca*, exhibit strong antibacterial and antioxidant properties [128].

Fungal polysaccharides, complex compounds synthesized by edible fungi and yeasts, possess promising antimicrobial characteristics. For instance, *Catathelasma ventricosum* carboxymethylated polysaccharides and *Grifola frondosa* SH-05 intracellular zinc polysaccharides display potent antibacterial activity [127]. Though the exact antibacterial mechanism remains partially understood, polysaccharides are believed to disrupt bacterial cell walls and membranes, leading to increased permeability and growth inhibition [112].

Polysaccharides inhibit microorganisms through multiple interrelated pathways. Cationic polysaccharides, such as chitosan, interact electrostatically with negatively charged microbial cell surfaces, leading to membrane disruption, increased permeability, and leakage of intracellular components. Other polysaccharides destabilize cell walls, interfere with nutrient transport, and impair intracellular metabolism, ultimately inhibiting growth or inducing cell death [129,130]. In addition, polysaccharides can inhibit microbial adhesion and biofilm formation or disrupt established biofilms, reducing microbial resistance on food contact surfaces [131].

Evaluation of fucoidan from *Spatoglossum asperum* against *Aeromonas hydrophila* demonstrated notable antibacterial efficacy. Similarly, polysaccharides from *Cystoseira barbata* showed inhibitory effects against foodborne pathogens such as *Staphylococcus aureus*, highlighting their potential applications in food preservation. Animal-derived polysaccharides, including those from cuttlefish and smoothhounds, possess high sulfate and uronic acid contents, indicating strong antibacterial and antioxidant potential. These polysaccharides may serve as natural preservatives in food manufacturing due to their anti-pathogenic activity and ability to enhance oxidative stability [70].

In some polysaccharide-based composite systems and active packaging materials, localized generation of reactive oxygen species may further contribute to antimicrobial stress and inhibition, acting synergistically with membrane-disruptive effects [124].

Polysaccharide-based coatings, films, and additives have demonstrated broad-spectrum antimicrobial activity against major foodborne pathogens, including *Escherichia coli*, *Staphylococcus aureus*, *Listeria monocytogenes*, and *Salmonella* spp., as well as spoilage fungi and yeasts. Their application in fresh produce, meat, dairy, and seafood systems has shown significant potential to delay spoilage and enhance food safety [132,133].

Antimicrobial efficacy is strongly dependent on polysaccharide molecular characteristics, including molecular weight, charge density, branching, and functional group composition. Lower molecular weight fractions often exhibit enhanced diffusion and microbial interaction, while higher cationic density improves binding to bacterial membranes. Structural modifications such as quaternization, sulfation, and controlled deacetylation have been shown to significantly enhance antimicrobial potency, underscoring the importance of structure–function relationships in designing effective polysaccharide-based preservatives [21,134].

### 4.3. Physicochemical Functions and Integrated Preservation Outcomes

In addition to their biological activities, polysaccharides contribute to food preservation through physicochemical mechanisms that directly influence food stability and quality. Their excellent film-forming and gel-forming abilities enable the development of edible coatings and biodegradable packaging materials that act as selective barriers against oxygen, moisture, and gas exchange. These barriers slow oxidative reactions, reduce dehydration, and limit microbial growth, particularly in fresh and minimally processed foods [21].

Polysaccharide matrices also function as effective delivery systems for controlled release of antioxidants, antimicrobials, and other bioactive compounds. Encapsulation within polysaccharide networks allows sustained release during storage, enhancing long-term preservation efficacy while minimizing sensory and nutritional impacts [14]. Furthermore, polysaccharides improve water-holding capacity, viscosity, emulsion stability, and textural integrity, preventing syneresis and maintaining desirable mouthfeel and appearance throughout shelf life [20].

**Figure 3:**
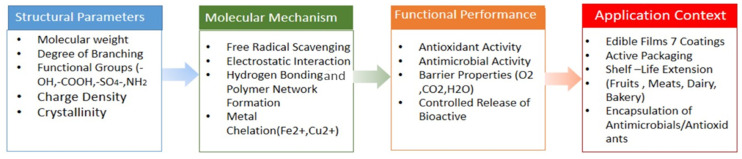
Conceptual figure illustrating active and barrier function of polysaccharide film.

Overall, the antioxidant, antimicrobial, and physicochemical functions of polysaccharides operate synergistically to delay spoilage, enhance food safety, and maintain quality. By simultaneously controlling oxidative degradation, microbial proliferation, and physicochemical instability, polysaccharides emerge as nature-derived, multifunctional guardians of freshness, supporting the development of sustainable, clean-label, and bio-based food preservation technologies. Figure 3 is a conceptual figure illustrating the active and barrier functions of polysaccharide film through a conceptual figure or schematic framework illustrating the following relationships: Structure → Molecular mechanism → Functional performance → Application context.

### 4.4. Mechanistic Aspects of Transport and Barrier Properties in Polysaccharide Films

The functional properties of polysaccharide-based active films are mainly explained by the following:

i- Diffusion-controlled transport,
ii- Permeability coefficients,
iii- Matrix densification,
iv- Humidity-induced plasticization effects.

These mechanisms are discussed below.

Mass transfer of gases and water vapor from polysaccharide films generally follows the solution diffusion transport. In this mechanistic way permeant molecules absorb into the matrix of polymer and diffuses into intermolecular free spaces and then desorb into the opposite surface side.

Permeability (P) is express as P=DXS

where D is the diffusion coefficient and S is the solubility coefficient.

Hydrophilic polysaccharides such as carrageenan, xanthan gum, chitosan, and plant mucilage have a high affinity for water molecules. This increases the solubility term (S) and consequently increases water vapor permeability (WVP), particularly under condition of high relative humidity. Therefore, barrier performance is not a single property of material but also the influence of environmental humidity [135].

Permeability coefficients and structure–barrier correlation is mainly based on barrier efficiency, which is mainly influence by structural parameters such as degree of crystallinity, polymer chain packing density, molecular weight, and extent of hydrogen bonding. Molecule mobility is reduced by higher crystalline systems, e.g., bacterial cellulose. These polysaccharides restrict diffusion pathways and cause lower diffusion coefficients (D) and make better and modified barrier properties. Thus, crystalline system and intermolecular interactions are main determinants of permeability coefficients and must be correlated with water vapor permeability or values of oxygen transmission rate [136].

Matrix Densification and Tortuosity Effects are governed by adding reinforcing agents, e.g., nanocellulose, polyphenols, essential oils, or inorganic nanofibrills that modify the microstructure of polysaccharide films by increasing hydrogen bonding interactions, crosslink density, and structural compactness. It results in increase of the tortuosity factor (τ), creating a more complicated and elongated diffusion pathway for permeant molecules.

$$\mathrm{The}\;\mathrm{effective}\;\mathrm{diffusion}\;\mathrm{coefficient}\;\mathrm{can}\;\mathrm{be}\;\mathrm{described}\;\mathrm{as}:\;Deff=\frac{D0}{\tau }$$

where D_0_ represents intrinsic diffusion and τ represents tortuosity. Increased tortuosity effectively reduces gas and moisture permeability [137].

The other parameter is humidity-induced plasticization and polymer relaxation to explain functional properties of active films. Polysaccharide-based films are highly sensitive to relative humidity due to their hydrophilic nature. Water molecules act as secondary plasticizers by disrupting intermolecular hydrogen bonds, increasing chain mobility and diffusion coefficients, and expanding free volume. As stated above, increasing the relative humidity, e.g., >75%, then water vapor permeability values also increase profoundly because of relaxation of the polymer network due to induced humidity. Thus, barrier evaluation must focus on environmental conditions when explaining permeability data [138].

**Figure 4:**
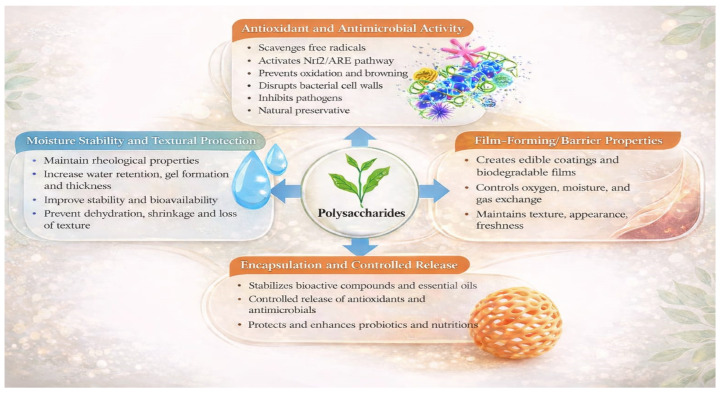
Multifunctional roles of natural polysaccharides in food preservation.

Figure 4 summarizes the key bioactivities and physicochemical functions through which polysaccharides act as multifunctional food preservatives, integrating oxidative protection, microbial inhibition, barrier formation, and quality stabilization.

## 5. Applications of Polysaccharides in Food Preservation

Polysaccharides have emerged as versatile and sustainable materials for food preservation due to their biodegradability, biocompatibility, and multifunctional properties. Their application spans edible films and coatings, shelf-life extension across diverse food systems, and delivery platforms for bioactive compounds, supporting clean-label and eco-friendly preservation strategies. The food matrix is often thickened by polysaccharides because they modify the rheological characteristics of the food matrix, increasing water retention and gel formation.

### 5.1. Edible Films and Coatings

Edible films and coatings based on polysaccharides play a crucial role in preserving food quality by acting as protective barriers and carriers of active agents. These films regulate moisture and oxygen transfer, thereby slowing dehydration, respiration, lipid oxidation, and microbial growth. Polysaccharides such as chitosan, alginate, pectin, carrageenan, and starch exhibit excellent film-forming capacity due to their hydrogen-bonding ability and polymeric network formation [21].

In addition to passive barrier functions, polysaccharide-based films serve as active packaging systems through the incorporation and controlled release of antimicrobial and antioxidant compounds. Bioactive such as essential oils, organic acids, phenolics, and plant extracts can be embedded within the polysaccharide matrix, enabling sustained release during storage and enhancing long-term preservation efficacy. Among these materials, chitosan is particularly valued for its intrinsic antimicrobial activity, while alginate and pectin provide superior moisture control and structural integrity. Carrageenan and starch-based films are widely used for their cost-effectiveness and compatibility with food matrices [20,21].

### 5.2. Shelf-Life Extension in Various Food Systems

Polysaccharides have been extensively applied across diverse food systems due to their ability to enhance safety, quality, and shelf stability through moisture regulation, oxidation control, and microbial inhibition. Their effectiveness has been demonstrated in fresh produce, meat and seafood, dairy systems, and bakery products, where they function as coatings, films, and functional additives.

#### 5.2.1. Fresh Fruits and Vegetables

In fresh fruits and vegetables, polysaccharide-based edible coatings play a critical role in delaying postharvest deterioration (Table 4). These coatings reduce respiration rate, moisture loss, and enzymatic browning while preserving firmness and sensory quality. Materials such as alginate, chitosan, cellulose derivatives, and pectin are widely used to form biodegradable barriers that limit oxygen and water vapour transfer, thereby slowing ripening and microbial spoilage [139,140].

For instance, alginate/cellulose nanofibril coatings applied to Andean blueberries significantly reduced respiration rate and moisture loss, while carrageenan/glycerol coatings on papaya delayed ripening, enhanced firmness retention, and minimized dehydration during storage [139]. These effects collectively contribute to extended shelf life and maintained nutritional quality.

#### 5.2.2. Meat and Seafood Products

Fresh meat and seafood provide favorable conditions for microbial growth and oxidative reactions, leading to rapid spoilage. Polysaccharide-based coatings effectively address these challenges by inhibiting lipid and myoglobin oxidation, suppressing microbial proliferation, and maintaining color and water-holding capacity (Table 5). Chitosan, alginate, and composite polysaccharide coatings have shown particular efficacy in extending refrigerated shelf life by reducing spoilage bacteria and oxidative deterioration [129,130]. Edible coating systems combining chitosan and hydroxypropyl methylcellulose (HPMC), supplemented with cellulase, have demonstrated improved lipid oxidation stability, pH control, reduced water activity, enhanced microbiological quality, and color stability in pork during cold storage. These coatings significantly reduced lipid oxidation over 14 days and extended shelf life by improving antimicrobial activity and overall product quality [162].

#### 5.2.3. Dairy Products and Beverages

In dairy products and beverages, polysaccharides function primarily as stabilizers and protective agents. They improve texture, prevent phase separation, and enhance microbial stability, while also protecting sensitive bioactive compounds and probiotic cultures in fermented dairy products and functional beverages. Through their water-binding and network-forming properties, polysaccharides contribute to both physical stability and extended shelf life in these systems [14].

#### 5.2.4. Bakery and Ready-to-Eat Foods

Bakery products, particularly bread, are highly susceptible to staling during storage, characterized by moisture redistribution, crumb hardening, and loss of freshness. Polysaccharides such as starch derivatives, cellulose ethers, and konjac glucomannan have been shown to improve moisture retention and delay starch retrogradation. The incorporation of konjac glucomannan (KGM) and its derivative, konjac superabsorbent polymer (KSAP), significantly slowed crumb hardening and amylopectin retrogradation by stabilizing gas cell structure during baking [198].

Thermogravimetric analysis revealed that KGM and KSAP reduced water loss and increased the proportion of compartmentalized water, leading to a more gradual release of moisture during storage. As a result, konjac-enriched bread exhibited a markedly reduced staling rate and improved shelf stability compared to control samples [198]. In ready-to-eat foods, polysaccharide-based films and coatings similarly enhance freshness and safety during storage and distribution.

### 5.3. Polysaccharides as Delivery Carriers

**Figure 5:**
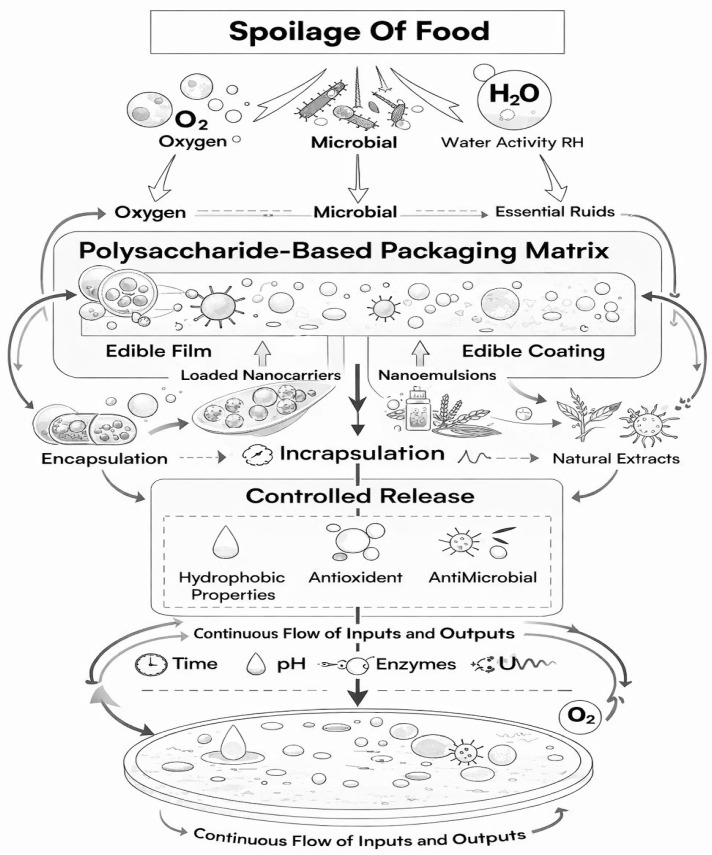
Stimuli-responsive nanostructured polysaccharide packaging for controlled bioactive release.

Beyond direct preservation, polysaccharides are increasingly employed as delivery carriers for bioactive compounds due to their structural flexibility and functional versatility. They are widely used for the encapsulation of essential oils, antioxidants, enzymes, probiotics, and other functional ingredients, protecting them from environmental degradation and enabling controlled release. Figure 5 is showing stimuli-responsive, nanostructured polysaccharide-based active packaging system with controlled bioactive release governed by environmental triggers and diffusion kinetics.

Micro- and nanoencapsulation systems based on polysaccharides such as alginate, chitosan, pectin, starch, and dextran have been extensively developed. These systems enhance the stability, solubility, and bioavailability of encapsulated compounds while minimizing sensory impacts. Encapsulation techniques include ionic gelation, spray drying, coacervation, and emulsion-based methods, which allow precise control over particle size, release kinetics, and functionality [14].

Polysaccharide-based nano-delivery platforms are particularly promising in active packaging and functional food applications, as they enable sustained antimicrobial and antioxidant release at the food surface, improving preservation efficiency while reducing the need for high additive concentrations [21].

**Figure 6:**
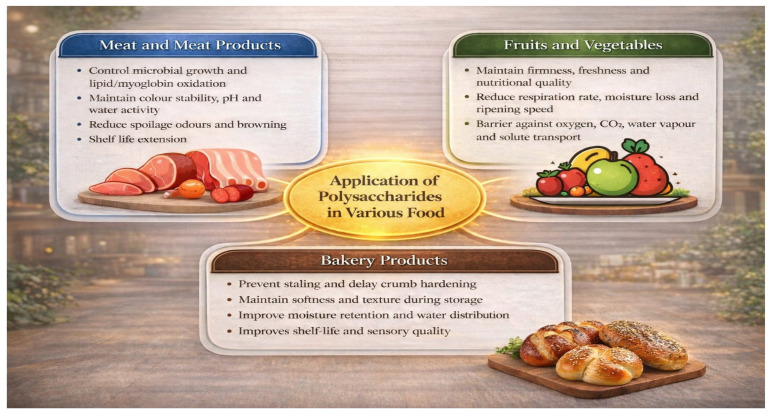
Applications of polysaccharides in different food systems and their roles in shelf-life extension [199].

Figure 6 illustrates the multifunctional role of polysaccharides in food preservation by controlling microbial growth and oxidation in meat products, reducing respiration rate and moisture loss in fruits and vegetables, and delaying staling while improving moisture retention in bakery products, ultimately contributing to enhanced shelf life and sensory quality.

## 6. Challenges and Limitations

Despite the growing interest in polysaccharides as natural alternatives to synthetic preservatives, several challenges and limitations restrict their widespread application in food preservation systems. These limitations arise from sensory impacts, economic and scalability constraints, regulatory complexities, stability issues in diverse food matrices, and inherent variability in natural polysaccharide composition.

One of the primary challenges associated with polysaccharide-based preservation systems is their potential to alter the sensory and textural attributes of foods. Edible coatings and films may affect surface appearance, gloss, firmness, mouthfeel, and flavour release, particularly when applied at higher concentrations or with inadequate formulation control. In fresh produce, excessive coating thickness can result in an undesirable waxy appearance or altered respiration behaviour, while in bakery and meat products, polysaccharides may influence texture, chewiness, or moisture perception. Such sensory deviations can negatively affect consumer acceptance, highlighting the need for formulation optimization that balances preservation efficacy with sensory quality [7,200].

Application of polysaccharide-based film from the lab to industries always remains challenging for economic and technical points of view. There is no doubt that the raw material of polysaccharides is abundant in nature, but its extraction, purification, and modification drive up the cost. Moreover, traditional methods of batch casting and solvent evaporation are not readily lead in these continuous manufactures. Transitioning to upgrade by extrusion, spray-coating, and roll-to-roll processing cause’s complex challenges in viscosity control, drying kinetics, and product uniformity [20,201].

Regulatory and food safety considerations also pose significant challenges. The regulatory status of polysaccharides varies depending on their source, degree of modification, and intended use as food additives, edible coatings, or packaging materials. In many jurisdictions, approval processes require extensive toxicological and migration data, and regulatory ambiguity may exist regarding whether a material classified as a food additive or a packaging component. Although regulatory considerations are sometimes viewed as administrative roadblocks, they actually serve as a strategic limitation that shapes the research, development, and commercialization routes for polysaccharide-based preservation systems. Contrary to synthetic preservatives, which have globally harmonized regulations and well-established safety profiles, polysaccharides fall into a regulatory gray area where functional claims and jurisdictions differ greatly. Compliance with regional food safety regulations and labelling requirements can delay product development and market entry, particularly for chemically modified or composite polysaccharide systems [21]. This multiplicity of classifications introduces structural uncertainty, particularly for chemically modified or composite systems incorporating antimicrobials or other bioactive agents. Manufacturers face variable and sometimes conflicting regulatory requirements across different markets, which increases development timelines, escalates R&D costs, and discourages investment in innovation.

Another important limitation is the poor stability of many polysaccharide systems in certain food matrices, particularly those with high moisture content. Due to their hydrophilic nature, polysaccharide-based films often exhibit high water vapor permeability and limited resistance to humidity, which can compromise their barrier performance and mechanical integrity during storage. In complex food systems such as emulsions, acidic beverages, or high-fat products, polysaccharide networks may swell, dissolve, or lose structural cohesion, reducing their preservative effectiveness. Addressing these stability issues remains a critical research focus, often requiring blending, crosslinking, or incorporation of hydrophobic components [14].

Variability in natural polysaccharide composition further complicates their application in food preservation. Polysaccharides derived from plant, algal, fungal, or animal sources often show significant batch-to-batch variation in molecular weight, monosaccharide composition, degree of substitution, and impurity levels. These variations directly influence functional properties such as solubility, viscosity, film-forming ability, and bioactivity, leading to inconsistent performance and challenges in quality control. Standardization of extraction methods, raw material sourcing, and analytical characterization is therefore essential to ensure reproducibility and industrial reliability [20]. Reproducibility and natural heterogeneity represent significant challenges in the development of polysaccharide-based films. The inherent diversity of natural polymers is a significant but frequently overlooked obstacle to the industrial adoption of polysaccharide-based preservation solutions. Polysaccharides originating from plants, algae, fungi, or animals show notable heterogeneity in molecular weight distribution, monosaccharide makeup, branching patterns, degree of substitution, and residual impurities, in contrast to synthetic preservatives with well-defined chemical structures. A number of variables, including species, growth circumstances, harvest time, and extraction technique, affect these variances [5]. Such intrinsic heterogeneity has a direct influence on physicochemical and functional properties—including chain entanglement, film-forming ability, mechanical strength, water vapor and gas barrier effectiveness, and interactions with incorporated bioactive agents leading to inconsistent performance across production batches. This variability can compromise quality control, process reproducibility, functional predictability, and regulatory compliance when polysaccharide materials are scaled up from laboratory to industrial settings. Addressing these issues requires systematic molecular characterization, standardized raw material specifications, and controlled extraction methodologies to ensure consistent structure–function relationships and reproducible performance in commercial applications [6]. Absence of standardized evaluation framework is another challenge. A persistent and systemic limitation in polysaccharide-based preservation research is the absence of universally accepted, standardized evaluation frameworks for performance benchmarking. Although numerous studies report improvements in mechanical strength, moisture barrier properties, and antimicrobial efficacy of polysaccharide films and coatings, the methodologies employed vary widely across investigations encompassing differences in test conditions, evaluation criteria, and analytical procedures [7]. For instance, barrier properties such as water vapor permeability and oxygen transmission rate are often measured under different relative humidity levels, film thicknesses, and temperature regimes, making a direct comparison between non-trivial studies [8]. According to this, other microbial targets and heterogeneous models (agar diffusion, viable plate counts, and vapor-phase tests) are used to evaluate antimicrobial activity; however, the lack of widely accepted test standards results in contradictory interpretations of efficacy.

**Figure 7:**
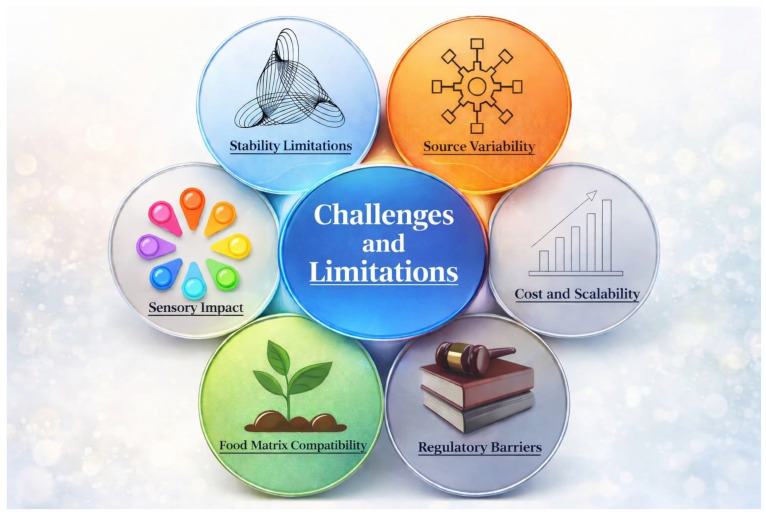
Challenges and limitations of application of polysaccharides.

Overall, while polysaccharides offer substantial potential as multifunctional, sustainable food preservation agents, overcoming these challenges is essential for successful commercialization. Future research should focus on improving material stability, reducing production costs, ensuring regulatory compliance, and minimizing sensory impacts through advanced formulation strategies and interdisciplinary approaches. Addressing these limitations will be critical to fully realizing the role of polysaccharides as nature-derived guardians of freshness in modern food systems (Figure 7).

## 7. Future Prospects and Emerging Trends

### 7.1. Scalability and Processing Constraints

The rapid evolution of food preservation technologies, coupled with increasing demand for sustainable and clean-label solutions, has positioned polysaccharides as key materials for future innovation. Emerging research trends emphasize the development of advanced functional systems that go beyond conventional preservation, integrating responsiveness, nanoscale engineering, and sustainability-driven design.

One of the most significant future directions is the development of smart, intelligent, and active packaging systems based on polysaccharides. These systems are design to interact dynamically with the food environment by responding to changes in pH, temperature, moisture, or microbial activity. Polysaccharide-based matrices are increasingly explored as carriers for freshness indicators, gas scavengers, and antimicrobial agents that can provide real-time information about food quality while simultaneously extending shelf life. The incorporation of natural dyes, redox-sensitive compounds, and biosensors into polysaccharide films has shown promise for intelligent monitoring of spoilage and safety, particularly in fresh and minimally processed foods.

### 7.2. Reproducibility and Raw Material Standardization

Nanopolysaccharides and nanocomposite biofilms represent another rapidly expanding area of research. The use of cellulose nanocrystals, nanofibrillated cellulose, chitosan nanoparticles, and starch nanostructures significantly enhances the mechanical strength, barrier properties, and functional stability of edible films and coatings. At the nanoscale, polysaccharides exhibit improved surface area and interaction with active compounds, enabling more efficient antimicrobial and antioxidant performance.

### 7.3. Standardization of Barrier and Functional Assays

The combined use of nanofilms with polysaccharides, along with their synergistic interaction of essential oils and antimicrobial proteins, have proved enhanced barrier properties. Nanocomposite biofilms have demonstrated superior resistance to oxygen and moisture transmission, making them particularly suitable for high-moisture and oxygen-sensitive food systems. From a broader perspective, polysaccharides are expected to play a critical role in advancing the circular bioeconomy within the food sector. The valorization of agro-industrial residues, food processing by-products, marine biomass, and microbial fermentation streams into functional polysaccharides represents a sustainable pathway for waste reduction and resource efficiency. By replacing petroleum-based packaging and synthetic preservatives, polysaccharide-based materials contribute to reduced environmental impact, improved biodegradability, and alignment with global sustainability and carbon-reduction targets.

**Figure 8:**
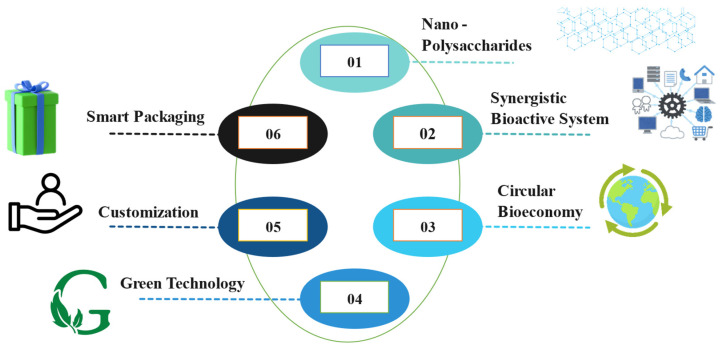
Future prospects and emerging trends.

Future preservation strategies are also increasingly focused on synergistic systems that combine polysaccharides with essential oils and antimicrobial peptides. Polysaccharides act as protective matrices that stabilize volatile or sensitive compounds, reduce sensory intensity, and enable controlled release during storage. Encapsulation of essential oils within polysaccharide-based micro- and nanostructures has been shown to enhance antimicrobial efficacy while minimizing flavor alteration (Figure 8).

#### Industrial Implementation and Economic Feasibility

The integration of polysaccharide utilization with green extraction and bioprocessing technologies is another emerging trend that aligns strongly with sustainability goals. Advanced extraction methods such as enzyme-assisted extraction, pulsed electric fields, subcritical water extraction, and fermentation-based production are being increasingly adopted to obtain high-purity polysaccharides with tailored functional properties. These environmentally friendly approaches reduce solvent consumption, energy demand, and processing time, while improving yield and structural consistency. Such advances support the scalable and sustainable production of polysaccharides for industrial food applications.

Overall, future research will likely focus on multifunctional, nanoenabled, and intelligent polysaccharide systems that integrate preservation performance with environmental responsibility. Continued advances in material engineering, green processing, and synergistic bioactive systems will further strengthen the role of polysaccharides as cornerstone materials in next-generation food preservation technologies.

## 8. Conclusions

Polysaccharides, as versatile natural macromolecules, serve as nature’s guardians of freshness in food preservation, owing to their structural diversity, functional versatility, and broad bioactivities. Derived from plants, animals, fungi, and microbes, these biopolymers exhibit key properties such as biodegradability, biocompatibility, and non-toxicity. Their chemical characteristics monosaccharide composition, molecular weight, branching, and glycosidic linkages directly influence antioxidant, antimicrobial, and stabilizing effects, which are critical for maintaining food quality and extending shelf life. In food systems, polysaccharides act as effective moisture and oxygen barriers in edible films and coatings, serve as carriers for bioactive compounds, and enhance textural and physicochemical stability across fruits, vegetables, meats, dairy, and bakery products. Polyphenol-enriched polysaccharide coatings further offer innovative solutions for active and intelligent packaging, combining preservation efficiency with environmental sustainability. Compared to conventional synthetic preservatives and packaging, polysaccharides provide safer, eco-friendly alternatives. While challenges such as natural variability, production costs, and regulatory considerations remain, advances in extraction, modification, and nanoengineering continue to expand their potential. By integrating intelligent packaging technologies and synergistic bioactive systems, polysaccharides promise fresher, safer, and more sustainable food systems that meet both consumer expectations and environmental goals.

## Figures and Tables

**Table 1:** Categories, polysaccharides types, and their chemical composition.

Category	Species/Raw Material	Polysaccharide Type	Composition/Monosaccharides	Family/Group	Key References
Plant-derived	*Abelmoschus esculentus*	Abelmoschus gum	Gal, Rha, Ara	Malvaceae	[44]
	*Albizia procera*	Albizia gum	Arabinose, galactose	Leguminosae	[45]
	*Aloe barbadensis*	Aloe mucilage	Glucomannans	Liliaceae	[46]
	*Basella alba*	Leaf mucilage	Arabinogalactans	Basellaceae	[47]
	*Anacardium occidentale*	Cashew gum	Galactose-rich polysaccharide	Anacardiaceae	[48]
	*Dillenia indica*	Dillenia gum	Pectic polysaccharide	Dilleniaceae	[49]
	*Trigonella foenum-graecum*	Fenugreek mucilage	Galactomannans	Fabaceae	[50]
	*Acacia arabica*	Gum Arabic	Arabinogalactan	Leguminosae	[51]
	*Cyamopsis tetragonolobus*	Guar gum	Galactomannan	Leguminosae	[52]
	*Cochlospermum gossypium*	Gum kondagogu	Complex acidic polysaccharide	Cochlospermaceae	[53]
	*Lannea woodier*	Gum odina	Polysaccharide gum	Anacardiaceae	[54]
	*Cordia obliqua*	Gum cordia	Galactomannan	Boraginaceae	-
	*Plantago ovata*	Ispaghula mucilage	Arabinoxylans	Plantaginaceae	-
	*Artocarpus heterophyllus*	Jackfruit starch	Amylose/amylopectin	Moraceae	[55]
	*Amorphophallus konjac*	Konjac glucomannan	Mannose, glucose	Araceae	[56]
	*Linum usitatissimum*	Linseed polysaccharide	Arabinoxylans	Linaceae	[57]
	*Ceratonia siliqua*	Locust bean gum	Galactomannan	Fabaceae	[48]
	*Moringa oleifera*	Moringa gum	Galactomannan	Moringaceae	[58]
	*Mimosa pudica*	Seed mucilage	Arabinogalactan-rich	Mimosaceae	[59]
	*Hibiscus esculentus*	Okra gum	Pectin + mucilage	Malvaceae	[50]
	*Solanum tuberosum*	Potato starch	Amylose/amylopectin	Solanaceae	[51]
	*Spinacia oleracea*	Leaf mucilage	Pectic polysaccharide	Amaranthaceae	[60]
	*Sterculia urens*	Karaya gum	Acidic polysaccharide	Sterculiaceae	[61]
	*Tamarindus indica*	Tamarind gum	Xyloglucan	Leguminosae	[62]
	*Terminalia randii*	Terminalia gum	Arabinogalactan	Combretaceae	-
Fungal-derived	*Aspergillus niger*	Chitosan	Glucosamine	Ascomycota	[63]
	*Aspergillus fumigatus*	Galactomannan	Gal, Man, Glc	Ascomycota	[64]
	*Beauveria bassiana*	Galactomannan	Gal, Man	Ascomycota	[65]
	*Lecanicillium muscarium*	Glucogalactan	Gal, Glc	Ascomycota	[66]
	*Lentinus edodes*	Lentinan	β-glucan	Basidiomycota	[67]
	*Ganoderma lucidum*	Heterogalactan, arabinoxyloglucan	Fuc, Gal, Glc	Basidiomycota	[68]
	*Grifola frondosa*	Grifolan	β-glucan	Basidiomycota	[69]
	*Aureobasidium pullulans*	Pullulan	Maltotriose units	Ascomycota	[70]
	*Poria cocos*	Pachyman	β-(1→3)-glucan	Basidiomycota	[71]
	*Pleurotus ostreatus*	Pleuran	β-glucan	Basidiomycota	[72]
	*Cryptococcus neoformans*	GXM	GlcA, Man, Xyl	Basidiomycota	[73]
	*Auricularia polytricha*	Xylomannan	Man, Xyl, Glc, Rib	Basidiomycota	[74]
	*Ganoderma tsugae*	Galactomannan	Gal, Man, Fuc, GlcN	Basidiomycota	[75]
	*Lecanicillium muscarium*	Fucogalactan	Fuc, Gal	Ascomycota	[76]
Marine algae	*Mastocarpus stellatus*	Carrageenan	Gal, Glc, Xyl, Man	Red algae	[77]
	*Chondrus armatus*	Carrageenan	Gal	Red	[78]
	*Ahnfeltiopsis flabelliformis*	Sulfated galactan	Gal, 3,6-AnGal	Red	[46]
	*Porphyra haitanensis*	Porphyran	Gal	Red	[79]
	*Gracilaria fisheri*	Sulfated galactan	Gal	Red	[80]
	*Alaria marginata*	Galactofucan	Fuc, Gal, Xyl	Brown algae	[81]
	*Cystoseira sedoides*	Fucoidan	Fucose, uronic acids	Brown	[82]
	*Eisenia bicyclis*	Laminaran	Glc	Brown	[83]
	*Sargassum fusiforme*	Laminaran	Glc, Gal	Brown	[84]
	*Laminaria japonica*	Laminaran	Man, Ara, Glc, Gal, Fuc	Brown	[77]
	*Codium divaricatum*	Sulfated galactan	Gal, Glc	Green algae	[84]
	*Capsosiphon fulvescens*	Ulvan	Rha, Xyl, Man	Green	[77]
	*Monostroma angicava*	Rhamnan sulfate	Rha	Green	[85]
Animal and Microbial	Shrimp/crab shells	Chitosan	GlcN	—	[39]
	Mammalian tissues	Hyaluronic acid	GlcA, GlcNAc	—	[86]
	*Xanthomonas campestris*	Xanthan gum	Glc, Man, GlcA	Microbial	[43]
	Bacterial fermentation	Bacterial cellulose	Glucose	Microbial	[40]

**Table 2:** Classification, structural, and functional aspects, quantitative/semi-quantitative metrics of polysaccharides.

Source Category	Species/Raw Material/Source	Key Structural Features	Functional Properties	Limitations/Variability	Quantitative/Semi-Quantitative Metrics	Structure–Performance Correlation for Active Film	References
Plant-derived Okra	Cellulose (nanocellulose, plant biomass)	Higher mucilage heteropolysaccharides, phenolic compounds, tocopherols, organic acids	Rich in Antioxidant profile for active film	Variation depends on strong genotype; effect of harvesting stage is also significant	Protein energy: **18.0–20.8% (small pods)** as compared to **15.1–18.9% (large pods);** antioxidant activity higher in immature pods; bioactive moieties (tocopherols, phenolics) vary profoundly among genotypes	Higher antioxidant potential; mucilage polysaccharides support film-forming ability and release of active compound	[92]
	Cellulose (nanocellulose from plant biomass)	Linear β-(1→4)-linked D-glucose chains; highly crystalline nanofibrils/nanocrystals with high aspect ratio	High mechanical strength, excellent barrier property, film-forming ability, biodegradability	Aggregation property; moisture sensitive; processing complexity	Tensile strength: up to **200–300 MPa (nanofibrils);** Young’s modulus: ~**100–150 GPa**; high crystallinity (>60%)	Linear crystalline, strong structure, intermolecular hydrogen bonding; nanofibrillar network, enhanced mechanical strength, reduced gas permeability	[93]
Plant-derived	Starch-based biopolymers/plasticized starch composites	Linear amylose and branched amylopectin polysaccharides; hydrogen-bonded network; plasticizer-induced amorphous structure	Biodegradable, film-forming, moderate barrier properties; improved flexibility with plasticizer	Moisture sensitivity; reduced strength at high plasticizer levels; variability in starch source	Tensile strength: **2–30 MPa;** Elongation: **20–80%;** WVP: **~2–6 × 10^−10^ g·m^−1^·s^−1^·Pa^−1^**	Amylose linear chains, plasticizer property disrupts hydrogen bonding, increased flexibility but lower strength; amorphous structure → enhanced extensibility	[94,95]
Plant/microbial-derived	β-glucan (from cereals such as oats, barley; also from fungi/yeast sources)	Linear β-(1→3) and β-(1→4) (cereals) or β-(1→3),(1→6) (fungal) glucan chains; high molecular weight polysaccharide	Thickening, viscosity enhancement, cholesterol-lowering, antioxidant, film-forming potential	Molecular weight variability; solubility depends on source and processing; viscosity loss during processing	Molecular weight: **50–2000 kDa;** viscosity (1%): **100–1000 cP**; β-glucan content in cereals: **4–11% (barley), 3–7% (oats),**	Linear β-linkages, strong intermolecular interactions; high MW, increased viscosity and film strength; branching (β-1,6), improved solubility and flexibility in films	[96]
Plant-derived	Nanocellulose (from lignocellulosic biomass)	Linear β-(1→4)-linked glucose chains; highly crystalline nanofibrils/nanocrystals; high aspect ratio	High mechanical strength, excellent barrier properties, biodegradability, reinforcement in films	Aggregation tendency; moisture sensitivity; processing challenges	Tensile strength: **200–300 MPa;** Young’s modulus: **100–150 GPa;** crystallinity: **>60%**	Crystalline nanofibrillar network, strong hydrogen bonding; enhances mechanical strength and reduces gas permeability	[97]
Plant-derived)	Pectin (fruit cell wall polysaccharide)	Anionic polysaccharide rich in galacturonic acid; degree of esterification-dependent structure	Film-forming, good oxygen barrier, antimicrobial carrier, biodegradable	High water sensitivity; poor moisture barrier; mechanical weakness without plasticizer	Tensile strength: 10–40 MPa; WVP: ~3–8 × 10^−10^ g·m^−1^·s^−1^·Pa^−1^; elongation: 10–50%	Carboxyl groups → ionic interactions and gel formation; degree of esterification controls film strength and permeability	[98]
Animal-derived	Chitosan (from crustacean shells)	Linear β-(1→4)-linked glucosamine; cationic polymer due to amine groups	Biodegradable, film-forming, moderate barrier properties; improved flexibility with plasticizer	Moisture sensitivity; reduced strength at high plasticizer levels; variability in starch source	Tensile strength: **2–30 MPa;** Elongation: **20–80%;** WVP:	Amylose linear chains, strength; plasticizer disrupts hydrogen bonding, increased flexibility but lower strength; amorphous structure, enhanced extensibility	[99]
Plant/microbial/animal-derived	Natural polysaccharides (starch, cellulose, chitosan, alginate, etc.)	Linear and branched polysaccharide chains; glycosidic linkages (α/β); varying molecular weights	Biocompatibility, biodegradability, film-forming, bioactivity (antioxidant, antimicrobial)	Source-dependent variability; sensitivity to moisture and pH; batch inconsistency	Not explicitly quantified	Chain structure (linear vs branched) and MW, influence viscosity, gelation, and film strength	[13]
Modified polysaccharides	Chemically/physically modified polysaccharides	Functional group substitutions (e.g., carboxyl, sulfate, acetyl); altered chain conformation	Enhanced solubility, bioactivity, and functional performance	Modification-dependent variability; structural degradation possible	Degree of substitution and molecular weight influence properties (no fixed values reported)	Functional group modification improves intermolecular interactions, enhancing film strength and functionality	[14,15]
Agro-industrial biomass	Polysaccharides from biomass residues (cellulose, hemicellulose, lignin)	Crystalline cellulose with amorphous hemicellulose and lignin matrix	Renewable, biodegradable, film-forming potential	Composition variability; extraction challenges	Cellulose: **30–50%; Hemicellulose: 15–35%; Lignin: 10–25%**	Crystalline cellulose enhances strength; amorphous components improve flexibility and processability	[16]
Biopolysaccharides	General polysaccharides (starch, cellulose, gums)	Linear/branched macromolecules with hydroxyl and carboxyl groups	Biodegradable, film-forming, thickening, stabilizing	Moisture sensitivity; variability in mechanical performance	Not explicitly reported	Functional groups and intermolecular interactions determine film strength, flexibility, and barrier properties	[17]
Plant/microbial/marine	Natural polysaccharides (cellulose, starch, pectin, alginate, chitosan, etc.)	Linear and branched polysaccharides; glycosidic linkages (α/β); diverse monosaccharide composition	Antioxidant, antimicrobial, thickening, gelling, stabilizing, film-forming	Source-dependent variability; sensitivity to environmental conditions (pH, temperature)	Not explicitly reported	Structural diversity and functional groups influence bioactivity, viscosity, and film-forming behavior	[20]
Polysaccharide-based carriers	Various polysaccharides (starch, gums, alginate, chitosan)	Polymer networks capable of encapsulation; hydrophilic matrix	Carrier and protector of bioactive compounds; controlled release; stabilization of additives	Interaction with food matrix; environmental sensitivity	Not explicitly reported	Network structure enables encapsulation and controlled release of active compounds in films	[19]
Plant/microbial/marine	Natural polysaccharides (cellulose, starch, pectin, alginate, chitosan, etc.)	Linear and branched structures; hydroxyl and carboxyl functional groups	Biodegradable, biocompatible, antioxidant, antimicrobial	Variability due to source and extraction method	Not explicitly reported	Functional groups and chain interactions govern film strength and bioactivity	[20]
Plant-derived	Bioactive polysaccharides	Complex heteropolysaccharides with branching and functional groups	Antioxidant, antimicrobial, immunomodulatory; delivery of bioactives	Structural complexity; variability among plant sources	Not explicitly reported	Structural complexity and branching enhance bioactivity and delivery efficiency in films	[21]
Plant-derived	Plant-based polysaccharides	Linear/branched carbohydrate polymers; hydroxyl-rich structure	Health-promoting effects, antioxidant activity, dietary fiber, stabilizing and thickening	Source variability; processing sensitivity	Not explicitly reported	Hydroxyl-rich structure enables hydrogen bonding, 111 affects viscosity and film stability	[22]
Animal-derived	Whey proteins (milk-derived biopolymers)			Moisture sensitivity; brittleness without plasticizer	Tensile strength and barrier properties discussed comparatively (no fixed universal values)	Protein network formation via intermolecular interactions enhances film strength and barrier properties	[23]
Plant-derived	*Epiphyllum oxypetalum* (arabinogalactan polysaccharide)	Arabinogalactan heteropolysaccharide (arabinose + galactose-rich chains)	Immunomodulatory, gut microbiota modulation, bioactive potential	Biological variability; extraction-dependent composition	Immunomodulatory effects demonstrated in vivo (no fixed physicochemical metrics for films)	Branched arabinogalactan structure supports bioactivity and potential incorporation into functional films	[24]
Plant-derived	Natural gums (gum arabic, guar gum, xanthan, etc.)	Branched heteropolysaccharides with hydrophilic functional groups	Film-forming, antioxidant, antimicrobial, stabilizing	Source variability; moisture sensitivity; mechanical limitations	Mechanical and barrier properties discussed comparatively (no fixed universal values)	Branched structures enhance flexibility; functional groups enable bioactive incorporation in films	[25]
Plant-derived	*Ceratonia siliqua* (carob; locust bean gum)	Galactomannan polysaccharide (mannan backbone with galactose branches)	Thickening, stabilizing, functional food ingredient, antioxidant potential	Composition depends on seed source and processing	Nutritional and compositional data discussed (no direct film metrics)	Galactomannan branching enhances viscosity and contributes to film-forming and stabilizing behavior	[26]
Plant-derived	*Linum usitatissimum* (*flaxseed mucilage*)	Heteropolysaccharide (arabinoxylans, rhamnogalacturonans)	Thickening, emulsifying, stabilizing, film-forming potential	Extraction method-dependent variability; composition differences	Extraction yield and rheological behavior discussed (no fixed universal values)	Heterogeneous structure contributes to viscosity and gel formation, supporting film development	[27]
Plant-derived	Edible gums (gum arabic, guar, karaya, etc.)	Complex branched polysaccharides with hydroxyl and carboxyl groups	Thickening, gelling, stabilizing, emulsifying, film-forming	Source variability; environmental sensitivity; batch inconsistency	Functional behavior discussed qualitatively (no fixed numeric values)	Functional groups enable hydrogen bonding and network formation, influencing film strength and stability	[28]
Marine-derived	Marine organisms (algae, seaweed polysaccharides)	Sulfated polysaccharides (fucoidan, carrageenan, alginate); complex heteropolysaccharides	Strong antioxidant activity, bioactivity, radical scavenging	Source and extraction variability; structural heterogeneity	Antioxidant activity reported (DPPH, ABTS)	Sulfate groups and polysaccharide structure enhance antioxidant activity and potential for active films	[29]
Fungal-derived	Fungal biopolymers (mycogenic polysaccharides)	Extracellular polysaccharides and biopolymer nanocomposites	Heavy metal adsorption, detoxification, bioactivity	Process-dependent variability; limited scalability	Adsorption efficiency discussed (no fixed numeric ranges for films)	Functional groups enable binding of metals; useful in active and functional coatings	[30]
Fungal-derived	Fungal chitin/chitosan	Linear β-(1→4)-linked N-acetylglucosamine chains	Biodegradable, antimicrobial, film-forming, biocompatible	Extraction complexity; variability in degree of deacetylation		Linear chains and amine groups enable strong film network and antimicrobial functionality	[31]
Fungal-derived	Mushroom β-glucans	β-(1→3), (1→6)-glucan structure with branching	Immunomodulatory, anti-inflammatory, bioactive	Species-dependent variability; extraction differences	Biological activity demonstrated (no film-specific quantitative values)	Branched β-glucan structure enhances bioactivity and incorporation into functional films	[32]
Fungal-derived	Mushroom-derived chitin/chitosan	Chitin and chitosan polysaccharides from mushrooms	Film-forming, antimicrobial, biodegradable packaging applications	Extraction yield variability; processing limitations	Extraction yield and characterization discussed (no fixed universal film values)	Polymer structure supports film formation and antimicrobial activity	[33]
Fungal-derived	Mushroom residues (polysaccharides, chitin)	Complex biopolymer matrix (chitin, glucans, proteins)	Sustainable packaging material, biodegradable films	Composition variability; processing challenges	Functional application discussed (no fixed numeric values)	Composite structure enhances film strength and sustainability	[34]
Marine-derived	Algal polysaccharides (fucoidan, alginate, carrageenan)	Sulfated heteropolysaccharid	Antioxidant, biomedical	Structural variability	Antioxidant activity (DPPH, radical scavenging discussed)	Sulfation enhances bioactivity and active film functionality	[35]
Marine-derived	Alginate	Linear copolymer (M/G blocks)	Gelation, film-forming	Ion sensitivity	Ca^2+^-induced gelation discussed	“Egg-box” model, strong gel networks	[36]
Marine-derived	Fucoidan, carrageenan	Sulfated polysaccharides	Antiviral, antioxidant	Sulfate variability	Bioactivity discussed	Sulfate groups, active film functionality	[38]
Animal/microbial	Chitosan	Linear β-(1→4) glucosamine	Antimicrobial, film-forming	pH sensitivity	Antimicrobial activity discussed	Cationic nature, microbial inhibition	[39]
Animal/microbial	Chitosan films	Polymer network	Active packaging	Mechanical limitations	Enhanced activity with additives	Additives improve film performance	[40]

**Table 3:** Functional properties of polysaccharide for preservation and application in food.

Biological Source	Polysaccharide	Key Functional Properties for Food Preservation	Application in Bioactive Packaging	Representative References
*Abelmoschus esculentus* (Okra pod)	Mucilage	Film-forming, Antioxidant, moisture barrier	Controlled-release systems and Edible coating	[92]
Plant biomass (wood/cotton)	Cellulose/Nanocellulose	Mechanical strength and structural stability	Biodegradable film reinforcement	[93]
Maize/Corn (*Zea mays*)	Starch	Film-forming, oxygen barrier	Biodegradable packaging	[94,95]
Plant-derived polysaccharides	Starch, Arabinoxylans (hemicellulose)	Film-forming ability, Gasses barrier (O_2_)	Edible films, antimicrobial carrier matrix, Biodegradable composites	[94,95]
Barley (*Hordeum vulgare*)	β-glucan	Antioxidant, viscosity enhancement, moisture control	Active coatings	[97]
Oats (*Avena sativa*)	β-glucan	Antioxidant potential, moisture retention	Edible coatings	[97]
Hardwood (e.g., Birch)	Xylan (hemicellulose)	Film-forming, oxygen barrier	Sustainable packaging	[96]
Citrus peel	Pectin	Gelling, antimicrobial synergy	Fruit & vegetable coatings	[98]
Brown seaweed (e.g., Laminaria)	Alginate	Gel formation, controlled release	Drug delivery systems, encapsulation matrices	[99]

**Table 4:** Application of polysaccharide-based coatings for postharvest preservation of fruits and vegetables.

Polysaccharide/Biopolymer	Coated Fruit/Vegetable	Target/Mechanism	Microbiological Stability	Chemical Stability	Physical Stability	Positive Effects	References
Alginate/nano-Ag	Shiitake mushroom (*Lentinula edodes*)	Fungal inhibition	Beneficial effect against fungal infection	↑ Reducing sugar and total soluble solids; ↓ electrolyte leakage	Retention of firmness and color	Shelf-life extended	[141]
Alginate/β-cyclodextrin/trans-cinnamaldehyde/pectin/calcium lactate	Watermelon (*Citrullus lanatus*)	Bacterial and fungal inhibition	Beneficial effect against bacterial and fungal infection	↑ Antioxidant activity and vitamin C	↓ Weight loss, softening, browning	Texture preserved; shelf-life extended	[142]
Alginate/ascorbic acid/citric acid	Mango (*Mangifera indica*)	Bacterial and fungal inhibition	Beneficial effect against bacterial and fungal infection	Retention of soluble solids	Retention of firmness, color, and pH; ↓ Weight loss	Shelf-life extension	[143]
Alginate/cellulose nanofibril (CNF)	Andean blueberry (*Vaccinium meridionale*)	Oxidative stress	Beneficial effect against bacterial and fungal infection	↓ Malondialdehyde and polyphenol oxidase	Retention of firmness and color	Maintained antioxidant stability	[144]
Alginate/chitosan (layer-by-layer)	Melon (*Cucumis melo*)	Fungal inhibition	Beneficial effect against fungal infection	—	Retention of firmness; ↓ respiration and water loss; prevented cell wall degradation	Delayed senescence	[145]
Alginate/rhubarb extract	Peach (*Prunus persica*)	Antibacterial (*Listeria monocytogenes*)	Beneficial antibactericidal effect	—	Retention of color	—	[146]
Carrageenan/glycerol	Papaya (*Carica papaya*)	Fungal inhibition	Beneficial effect	—	↓ Moisture loss; delayed ripening; firmness retained	Extended shelf-life	[147]
Carrageenan/ascorbic acid/citric acid/oxalic acid/CaCl_2_	Apple (*Malus domestica*)	Enzymatic and oxidative control	—	Controlled ethanol accumulation; reduced oxidative deterioration	↓ Weight loss and ethylene; retention of firmness and color	Delayed tissue degradation	[148]
Agar/κ-carrageenan/konjac glucomannan	Spinach (*Spinacia oleracea*)	—	—	—	↓ Weight loss and respiration rate; firmness retained	Freshness maintained	[60]
Agar/chitosan/acetic acid	Garlic (*Allium sativum*)	—	—	—	Retention of color; ↓ respiration	Quality maintained	[149]
Chitosan	Figs/Strawberries	Fungal inhibition	Growth of Alternaria alternata inhibited	Antioxidant capacity preserved; titratable acidity and pH retained	Color and texture retained	Extended shelf-life	[150]
Chitosan	Banana (*Musa* spp.)	Fungal inhibition (*Colletotrichum musae*, *Fusarium* spp.)	Beneficial effect	Retention of soluble solids and titratable acidity	Retention of firmness and peel color; delayed ripening	Extended shelf-life	[151]
Chitosan	Peach (*Prunus persica*)	Fungal inhibition (Aspergillus niger)	Beneficial effect	↑ Titratable acidity and vitamin C	Retention of firmness; ↓ respiration and ethylene	Delayed ripening and senescence	[152]
Chitosan/Laurus nobilis extract	Strawberry (Fragaria × ananassa)	Fungal and bacterial inhibition	Beneficial effect	Retention of soluble solids, titratable acidity, pH	↓ Senescence, weight loss and respiration; firmness and color retained	Quality maintained; extended shelf-life	[150]
Chitosan/chlorogenic acid	Papaya (*Carica papaya*)	Fungal and bacterial inhibition	Beneficial effect	Retention of L-ascorbic acid; ↓ ROS	↓ Weight loss and respiration; firmness retained	Shelf-life extended	[147]
Chitosan/arabic gum/moringa leaf extract	Garlic (*Allium sativum*)	Fungal inhibition	Beneficial effect	Retention of ascorbic acid	↓ Weight loss and respiration; inhibition of ethylene	Shelf-life extended	[149]
Chitosan/pomegranate peel extract (PPE)	Capsicum (*Capsicum annuum*)	Microbial cell wall	PPE phenolics + chitosan-alginate matrix	Retention of ascorbic acid	Retained weight, firmness and color	Inhibited microbial growth; extended shelf-life ~25 days	[153]
Chitosan + cinnamon oil	Sweet cherry (*Prunus avium* L.)	Cell wall and membrane	Antimicrobial; fungal inhibition	—	↓ Respiration; ↑ CO_2_; controlled decay	Shelf-life extended	[154]
Aloe vera gel + basil seed mucilage	Apricot (*Prunus armeniaca*)	Fruit metabolism and microbial growth	Phenolic accumulation; microbial inhibition	↓ Weight loss and soluble solids	↓ Respiration and ethylene; firmness retained	Delayed ripening and quality maintained	[155]
Aloe vera gel (AVG)	Grapes, tomatoes, peach, sweet cherry, litchi	Fungal phospholipid bilayer	Disrupts fungal membranes	Retains phenolics, flavonoids, antioxidant activity	↓ Respiration and ripening; firmness maintained	Quality and shelf-life preserved	[156]
Guar gum + ginseng extract (GSE)	Sweet cherry (*Prunus avium* L.)	Oxidative and enzymatic activity	—	↓ Oxidation; retains phenolics and ascorbic acid	Controlled water loss; firmness retained	Shelf-life extended ~8 days	[154]
Alginate + chitosan + ZnO nanoparticles	Guava (*Psidium guajava* L.)	Fungal and microbial inhibition	ZnO inhibits Phyllosticta psidicola	—	Prevented fruit rot; delayed maturation	Shelf-life extended from 7 to 20 days	[157]
Sodium alginate + chitosan + dietary fibers	Blueberry (*Vaccinium sect. Cyanococcus*)	Microbial cell membrane	Chitosan antimicrobial effect enhanced by fibers	↑ Phenolics, antioxidants	↓ Gas exchange and water loss; firmness retained	Mould and off-odor prevented; sensory shelf-life ↑ ~6 days	[158]
Alginate + chitosan + carrageenan	Fresh-cut lettuce (*Lactuca sativa*)	Enzymatic browning and oxidative stress	Suppresses ROS; inhibits PPO, PLD, LOX	↑ Antioxidant enzyme activity	↓ MDA content; delayed senescence	Quality maintained	[159]
Polysaccharide from Oudemansiella radicata	Shiitake mushroom	Gas exchange and enzymatic activity	—	Delays enzymatic degradation; retains volatiles	↓ Weight loss; firmness and microstructure retained	Extended shelf-life and metabolic stability	[141]
Alginate/pectin/calcium lactate	Fresh-cut watermelon	—	—	—	Texture preserved	Shelf-life extended	[142]
Xanthan/alginate/gellan gum	Fresh-cut jackfruit bulbs	Microbial inhibition	Growth of bacteria suppressed	—	—	Shelf-life extended	[160]
Cactus pear mucilage	Fresh-cut kiwifruit	—	—	—	Visual quality and flavor improved	Shelf-life extended	[161]
Carboxymethylcellulose (CMC)	Fresh-cut apples	Oxidative enzymes	—	Vitamin C and antioxidant capacity maintained	—	Quality enhanced	[148]

**Table 5:** Application of polysaccharides and biopolymeric coatings in meat and fish products: bioactive compounds, preservation effects, and outcomes.

Polysaccharide/Biopolymer Source	Food Product	Applied Bioactive Compound/Level of Addition	Preservation Effects/Results	References
Brown alga Cystoseira compressa (CCPS)	Minced beef	1× MIC, 2× MIC, 4× MIC	↓ TBARS (<2 mg MDA/kg), ↓ carbonyl content, stabilized heme iron, inhibited microbes, maintained MetMb <40%, strong antioxidant and antibacterial activity	[163]
Morinda officinalis polysaccharides (MOP)	Broiler breast meat	500 mg/L	Restored meat metabolites, reduced oxidative damage, improved meat quality	[164]
Polysaccharides from Malcolmia triloba (PSMT)	Ground beef	0.5%, 1%, 2%	↓ TBARS, inhibited MetMb, inhibited microbial proliferation, improved color stability, pH, moisture, and heme iron	[165]
Polysaccharides from green seaweed Chaetomorpha linum	Beef sausage	0.05%, 0.125%, 0.25%	↓ microbial load, ↓ lipid oxidation, improved pH, moisture, color, stabilized MetMb and heme iron	[166]
Postbiotics (Saccharomyces cerevisiae var. boulardii) + Malva sylvestris mucilage	Lamb meat	MSM + 10% PSB	↓ microbial growth, extended shelf life (>10 days), maintained moisture, pH, texture, inhibited lipid oxidation, preserved sensory quality	[167]
Sulfated exopolysaccharides from *Porphyridium cruentum*	Chopped/minced beef	0.5%, 1%, 2% (MIC–4×MIC against *L. monocytogenes*)	↓ protein and lipid degradation, inhibited spoilage bacteria, extended shelf life	[168]
Pistachio hull polysaccharides (PHCP)	Chopped/minced beef	0.5%, 1%, 2%	↓ TBARS, improved color stability	[169]
Heteropolysaccharide from *Lobularia maritima*	Raw minced beef	0.15%, 0.3%, 0.6%	↓ lipid oxidation, ↓ MetMb formation, strong antibacterial activity, improved shelf life	[170]
Blackberry polysaccharide	Chicken breast meat	1 g/kg, 3 g/kg (24 h marination)	Improved texture, ↑ water retention, ↓ volatile compounds, improved flavor	[171]
Water-soluble polysaccharide from Hammada scoparia leaves (PSP)	Minced beef	0.5%, 1%, 2% (vs. 0.5% BHA)	↓ lipid oxidation, ↓ microbial growth, maintained color, pH, moisture, improved shelf life and safety	[172]
Linseed water-soluble polysaccharide (LWSP) from *Linum usitatissimum*	Beef sausages (collagen casing)	0.125% LWSP; 0.062% LWSP + 0.062% ascorbic acid	Improved texture and sensory quality, retarded lipid oxidation, stabilized oxymyoglobin, natural alternative to ascorbic acid	[166]
Polysaccharides from garlic straw (GSP)	Minced beef	2% and 4%	Protected against lipid peroxidation, ↑ shelf life, improved color	[173]
Exopolysaccharide from Lactobacillus sp. Ca6 (EPS-Ca6)	Beef sausage	0.0625% + vitamin C 0.0625%, or EPS-Ca6 0.125%	Retarded lipid peroxidation, ↓ oxymyoglobin oxidation	[168]
Polysaccharides from Trigonella foenum-graecum (FWSP)	Beef sausage	0.05%, 0.125%, 0.25%	↓ lipid oxidation, inhibited myoglobin oxidation	[174]
Spirulina platensis polysaccharides (SPP)	Chinese-style pork sausages	0.1%, 0.25%, 0.5%	Prevented sensory deterioration, ↓ lipid peroxidation	[175]
Sodium alginate	Cold-smoked salmon	Sodium lactate and sodium diacetate	↓ *Listeria monocytogenes* growth	[176]
Sodium alginate	Bighead carp fillets	Horsemint essential oil	↓ lipid oxidation (TBA, TVN, peroxide, FFA), ↓ total viable and psychrotrophic counts	[176]
Sodium alginate + chia gum	Fresh meat	Protein hydrolysate of rainbow trout roe	↓ total viable counts (~3 log), ↓ lipid oxidation (TBA, peroxide, TVN)	[177]
Sodium alginate	Fresh chicken breast fillets	Propionic acid and thyme essential oils	↓ total viable counts (~4 log), ↓ lipid oxidation (TBA, 3 days)	[177]
Sodium alginate + maltodextrin	Chevon sausages	*Asparagus racemosus* extract	↓ lipid oxidation (TBA, FFA), ↓ total plate, psychrophilic, yeast and mold counts	[178]
Sodium alginate	Chicken breast	Bacteriophage φIBB-PF7A	Inhibited Pseudomonas fluorescens	[177]
Chitosan (75–90% DD) + fish collagen	Red porgy meat	—	↓ lipid oxidation (TVN, K values)	[179]
Chitosan (>90% DD)	Fresh lion pork	Gallic acid	↓ total viable counts, ↓ TBA, ↓ protein oxidation	[180]
Chitosan (>90% DD) + gelatin	Fresh pork	Nisin + grape seed extract	↓ lipid oxidation (TBA)	[180]
Chitosan (85% DD)	Silver carp	—	↓ total viable counts, ↓ lipid oxidation (TVN, TBA)	[181]
Chitosan	Red drum fillets	Grape seed extract + tea polyphenols	↓ lipid oxidation (TVN, TBA), ↓ total viable counts	[182]
Chitosan (>90% DD) + salmon fish bone gelatin	Fresh salmon fillet	Gallic acid + clove oil	↓ oxidation (TBA, peroxide), ↓ lipid oxidation, ↓ total viable counts	[183]
Maize starch	Chicken breast fillets	Grape juice	↓ total aerobic mesophilic, psychrophilic, Enterobacteriaceae, ↓ TBA (1.07 mg MDA/kg)	[184]
Starch	Chicken meat	Torch ginger inflorescence essential oil	↓ coliform growth, ↓ lipid oxidation (TBA)	[185]
Corn starch	Raw beef	Clove and cinnamon essential oils	↓ microbial populations	[186]
Cassava starch	Ground beef	Lemongrass essential oil	↓ total microbial counts	[187]
Cassava starch	Film development	Chitosan + gallic acid	Antimicrobial film with strong application for preservation of food	[188]
Corn starch–chitosan	Fresh beef slices	Pomegranate peel extract + Thymus kotschyanus EO	↓ total microbial and lactic acid bacteria count, ↓ lipid oxidation (TBA)	[189]
Cassava starch/gelatin	Sliced pork meat	Quercetin + TBHQ	↓ redness loss during 12 days	[188]
Ginger starch	Ground beef	Coconut shell liquid smoke	↓ (1.33 log), ↓ lipid oxidation (TBA)	[165]
Pectin	Pork loin	Oregano essential oil + resveratrol (nanoemulsion, 53.09 nm, PDI 0.21)	↓ lipid oxidation (TBA), ↓ protein oxidation	[190]
Sodium caseinate	Chicken breast fillet	Ginger essential oil (nanoemulsion, 57.4 nm, PDI 0.22, ζ −18.7 mV)	↓ total aerobic psychrophilic bacteria (6% ginger essential oil, 12 days)	[185]
Chitosan	Turkey meat	*Zataria multiflora* Boiss + Bunium persicum Boiss essential oils (nanoemulsion, 130.2 and 154.26 nm, PDI 0.28 and 0.24)	↓ total viable bacteria, psychrophilic bacteria, Pseudomonas, Enterobacteriaceae, lactic acid bacteria, yeasts and molds	[191]
Basil seed gum	Chicken fillets	Shirazi thyme + summer savory essential oils	↓ lipid oxidation (TBA, TVN, peroxide), ↓ total viable, psychrotrophic, lactic acid bacteria count	[192]
Glucomannan/carrageenan	Chicken meat	Camellia oil	↓ total viable counts (2.5–3.5% camellia oil, 10 days), ↓ lactic acid bacteria (3–3.5% camellia oil), ↓ lipid oxidation (TBA, TVN)	[193]
Alginate	Turkey fillets	*Trachyspermum ammi* essential oil (nanoemulsion, 73.5 nm, PDI 0.441)	Prevention of *Listeria monocytogenes* growth	[191]
Gelatin + Chitosan	Ready-to-eat carbonado chicken	Rosemary extract + ε-poly-L-lysine (nanoemulsion, 257 nm; coarse 1122.44 nm, ζ 32.5 and 26.25 mV)	↓ total microbial counts, ↓ yeasts and molds, ↓ TBARS (mg MDA/Kg) (16 days)	[183]
Chicken bone gelatin + Chitosan	Ready-to-eat chicken patties	Cinnamon essential oil + rosemary extract (nanoemulsion 183.6 nm; coarse 1370.83 nm, ζ 33.76 and 25.76 mV)	↓ total viable counts, ↓ TBARS (mg MDA/Kg) and TVN	[194]
Chitosan	Salmon fillets	Nisin + carvacrol (particle size 1741–3893 nm, PDI 0.095–0.675, ζ 47–54.77 mV)	↓ total viable counts, ↓ TBARS (mg MDA/Kg) and TVN (15 days)	[183]
Bream fish	Bream fish	Glycerol, vitamin C, tea polyphenols	Bacterial growth inhibited, sensory values enhanced	[195]
Cheese	Cheese	Glycerol, sorbitol, corn oil	Shelf-life extended	[196]
Poached turkey	Poached turkey	Nisin, novagard CB1, Guardian NR100, sodium lactate, sodium diacetate, potassium sorbate	*Listeria monocytogenes* growth inhibited	[197]

## Data Availability

No new data were created or analyzed in this study. Data sharing is not applicable to this article.
